# Commentary: Senescence-driven osteonecrosis of the femoral head in the elderly—A distinct pathophysiological entity

**DOI:** 10.3389/fmed.2026.1794073

**Published:** 2026-04-01

**Authors:** Stuart B. Goodman, Chase W. Smitterberg, Lynne C. Jones, Michael A. Mont

**Affiliations:** 1 Department of Orthopaedic Surgery, Stanford University School of Medicine, Redwood City, CA, United States; 2 The Rubin Institute for Advanced Orthopedics, Sinai Hospital of Baltimore, Baltimore, MD, United States; 3 Johns Hopkins Medicine Department of Orthopaedic Surgery, Baltimore, MD, United States

**Keywords:** geriatric, osteonecrosis, osteonecrosis of the femoral head (ONFH), osteoporosis, senescence, subchondral insufficiency fracture

## Introduction

We read with interest the article titled: “Senescence-driven osteonecrosis of the femoral head in the elderly—a distinct pathophysiological entity,” by Tong-jie Yang and co-authors (1). Based on vascular aging, marrow obesity, inflammation, and decreased mechano-adaptation, the authors suggest “senescence-driven osteonecrosis of the femoral head” as a unique disease entity in geriatric patients. We feel that the article greatly overextends its conceptual framework by redefining well-described clinical entities without enough clinical, radiological, epidemiologic, or mechanistic confirmation, despite the admirable goal of integrating gerontology research into musculoskeletal pathology.

## Discussion

The manuscript's frequent confusion of subchondral insufficiency fracture of the femoral head (SIFFH), known as spontaneous osteonecrosis of the knee (SPONK) when it occurs in the knee, with classic osteonecrosis of the femoral head (ONFH) is one of its main flaws. The underlying pathophysiology of both disorders is fundamentally different: SIFFH/SPONK is mechanical, fracture-driven, and frequently secondary ischemic, while ONFH is ischemic in origin (2–4). Although this distinction is acknowledged, the manuscript conceptually compresses it in effect, renaming insufficiency fractures as osteonecrosis.

The authors run the danger of wrongly diagnosing a fracture-induced osteoporotic collapse as “osteonecrosis.” The ONFH is not solely defined by histologic necrosis, which is a downstream finding in many musculoskeletal diseases (5, 6). Necrotic trabeculae in older patients often indicate secondary ischemia after subchondral fracture rather than primary vascular failure (7–9). The article makes a crucial conceptual mistake by treating necrosis as causal rather than consequential on multiple occasions.

The existence of a new disease category is not supported by the presented epidemiologic data. Rather, they reflect quickly destructive arthrosis in older patients and under-recognition and incorrect diagnosis of SIFFH. The manuscript's own cited series are small, retrospective, and varied, which limits their capacity to support a redefinition, and the rarity of idiopathic ONFH in patients over 60 years has been repeatedly shown (6, 10–12).

The imaging parts minimize distinguishing features and selectively highlight overlap. In contrast to ONFH, SIFFH and rapidly destructive arthrosis are characterized by the lack of a sclerotic demarcation line, significant marrow edema, irregular low-signal bands, and rapid joint destruction (10, 13–16). Although these characteristics have already been extensively documented in fracture-driven disease in osteoporotic bone, they are falsely offered as proof of a distinct senescence-driven osteonecrosis phenotype.

Instead of using information unique to a particular disease, the discussion of endothelial senescence, inflammation, and marrow adiposity mainly depends on extrapolation from general aging biology. Although there is little proof that these processes independently cause femoral head ischemia in geriatric people, they certainly have an impact on bone health. As a result, the suggested model continues to generate hypotheses rather than provide an explanation.

Senescence-driven diseases are thought to be indicated by the presence of senescence markers [e.g., p16, p21, and senescence-associated secretory phenotype (SASP) factors] in necrotic bone. Senescent cells, however, build up in almost all chronically damaged tissues, especially in older people. Their existence may merely represent the tissue's reaction to damage, ischemia, or fracture and does not prove predominance in etiology.

The study suggests biologic treatments, vasculoprotective medicines, and senolytics as sensible interventions. Clinical evidence in ONFH, SIFFH, or senior hip collapse does not support these hypothetical recommendations. More importantly, given the article's framing, they divert attention from the well-established principle that early mechanical unloading and fracture identification are crucial in older patient hip disease.

The paper runs the danger of promoting unsuitable treatment approaches by classifying SIFFH-like pathology as osteonecrosis. Weight-bearing restriction or fracture-specific therapies may be postponed if insufficiency fractures are treated as ischemic necrosis, which could aggravate results. Therefore, terminology precision is clinically consequential rather than conceptual.

It is reasonable to wonder what degree of evidence would be needed to support such a claim for a new senescence-driven entity, even though the current commentary contends that the available data are insufficient to support a new medical category. Generally speaking, convergent data from the epidemiologic, imaging, and mechanistic domains is necessary to define a unique pathophysiological entity, especially one that would change diagnosis and treatment. From an epidemiologic perspective, this would necessitate population-level data showing a repeatable clinical phenotype in older adults that cannot be attributed to SIFFH, osteoporotic collapse, incorrectly classified osteoarthritis, or established forms of osteonecrosis, with a unique incidence, natural history, and outcome profile verified in large registries or prospective cohorts as opposed to small, retrospective series with inconsistent diagnostic definitions. Imaging evidence would need to show dependable, prospectively identifiable radiographic or magnetic resonance imaging features that distinguish this proposed entity from fracture-driven disease before collapse, with acceptable interobserver agreement. Overlapping findings, such as rapid joint destruction, irregular subchondral signal changes, absence of a sclerotic demarcation line, or marrow edema, are well-established characteristics of rapidly destructive arthrosis and insufficiency fracture and cannot, by themselves, support disease reclassification.

Mechanistic or molecular evidence would need to show etiologic specificity, including that senescence-associated markers such as p16 or p21 expression or SASP factors precede structural failure and predict disease development independently of age, fracture, or ischemic injury; the presence of such markers in necrotic or damaged bone, particularly in geriatric patients, more plausibly reflects generalized aging or injury responses rather than disease-defining causality.

Until such data emerges in all of these categories, reframing existing fracture-driven or degenerative processes as a distinct osteonecrosis subtype risks conceptual confusion and clinical misdirection.

In conclusion, the paper does not persuasively establish “senescence-driven osteonecrosis” as a separate pathophysiological entity, despite offering a careful synthesis of aging biology and bone fragility. Instead of putting forth a novel osteonecrosis subtype, the work would be reinforced by rephrasing its premise to highlight aging-related susceptibility to insufficiency fracture with necrotic bone. We feel that the paper is thought-provoking, but conceptually overreaching, as stated, and its main assertions outweigh the supporting data.
